# 5-HT3 Receptors in Rat Dorsal Root Ganglion Neurons: Ca^2+^ Entry and Modulation of Neurotransmitter Release

**DOI:** 10.3390/life12081178

**Published:** 2022-08-02

**Authors:** Katiuscia Martinello, Antonietta Sucapane, Sergio Fucile

**Affiliations:** 1 IRCCS Neuromed, Via Atinense, 86077 Pozzilli, Italy; sergio.fucile@uniroma1.it; 2 Department of Physiology and Pharmacology, Sapienza University of Rome, P.le Aldo Moro, 5, 00185 Rome, Italy; antonietta.sucapane@uniroma1.it

**Keywords:** serotonin, fractional Ca^2+^ current, sensory neurons

## Abstract

Rat dorsal root ganglion (DRG) neurons express 5-hydroxytryptamine receptors (5-HT3Rs). To elucidate their physiological role in the modulation of sensory signaling, we aimed to quantify their functional expression in newborn and adult rat DRG neurons, as well as their ability to modulate the Ca^2+^-dependent neurotransmitter release, by means of electrophysiological techniques combined with fluorescence-based Ca^2+^ imaging. The selective 5-HT3R agonist mCPBG (10 μM) elicited whole-cell currents in 92.5% of adult DRG neurons with a significantly higher density current than in responding newborn cells (52.2%), suggesting an increasing serotoninergic modulation on primary afferent cells during development. Briefly, 5-HT3Rs expressed by adult DRG neurons are permeable to Ca^2+^ ions, with a measured fractional Ca^2+^ current (i.e., the percentage of total current carried by Ca^2+^ ions, Pf) of 1.0%, similar to the value measured for the human heteromeric 5-HT3_A/B_ receptor (P*_f_* = 1.1%), but lower than that of the human homomeric 5-HT3_A_ receptor (P*_f_* = 3.5%). mCPBG applied to co-cultures of newborn DRG and spinal neurons significantly increased the miniature excitatory postsynaptic currents (mEPSCs) frequency in a subset of recorded spinal neurons, even in the presence of Cd^2+^, a voltage-activated Ca^2+^ channel blocker. Considered together, our findings indicate that the Ca^2+^ influx through heteromeric 5-HT3Rs is sufficient to increase the spontaneous neurotransmitter release from DRG to spinal neurons.

## 1. Introduction

Serotonin (5-hydroxytryptamine; 5-HT) exerts multiple effects on sensory signaling [1] and is released from platelets, mast cells, and basophils following the injury of peripheral tissue [2], or from terminals of descending bulbospinal neurons in the dorsal horn of the spinal cord [3]. The role played by 5-HT in the descending control of pain (thoroughly reviewed by [4]) is extremely complex, as different 5-HT receptor subtypes expressed in distinct neuronal populations may alternatively contribute to descending facilitation or inhibition during development [5]. In particular, 5-HT interacts with a variety of 5-HT receptors present in the nerve endings of dorsal root ganglion (DRG) neurons [6], including G-protein-coupled 5-HT receptors and ionotropic 5-HT3 receptors (5-HT3Rs) [7,8,9], the latter belonging to the nicotinoid superfamily of excitatory ligand-gated ion channels [10,11]. In the last two decades, the role in the modulation of pain played by 5-HT3Rs expressed in sensory neurons has been extensively studied, yielding partly contradictory results, with selective agonists producing both anti- and pro-nociceptive effects in different experimental conditions (for review see [12]). The genetic deletion of 5-HT3Rs in mice led to a marked decrease in the formalin-induced nociceptive responses, confirming their involvement in pain modulation [13]. The 5-HT3R is composed of five subunits, assembled in a ring surrounding a central cation-selective ionic pore. To date, two 5-HT3R subunits, 5-HT3_A_ and 5-HT3_B_, have been cloned and characterized, and genes of putative 5-HT3_C_-3_E_ subunits have been described [14]. The functional properties of homomeric 5-HT3_A_Rs differ from those of heteromeric 5-HT3_A/B_Rs [15,16,17], with increased single-channel conductance and decreased relative Ca^2+^ permeability when 5-HT3_A_ and 5-HT3_B_ subunits are co-expressed [15]. The expression of 5-HT3Rs with specific subunit compositions in different regions of the nervous system and different subcellular locations [5,18] may reflect their distinct physiological role. The presynaptic location of 5-HT3Rs is responsible for the Ca^2+^ increase in nerve terminals in several CNS regions such as the striatum, hippocampus, amygdala, and cerebellum [19,20]. Thus, 5-HT facilitates the release of a variety of neurotransmitters such as dopamine in the striatum and olfactory tuberculum [21], GABA in the nucleus of the solitary tract, hippocampus, amygdala, and spinal cord [22,23,24,25], and glutamate in the dorsal vagal motor nucleus [26]. Although several studies described the properties of 5-HT3Rs expressed by DRG neurons [4,8,27,28,29] and by primary afferent fibers impinging on the superficial layers of the dorsal horn [30], conflicting data have been documented about their exact functional role: they may enhance the release of glutamate and substance P, explaining the observed electrophysiological and behavioral evidence of increased 5-HT3-mediated nociception (see for review [4]), but also inhibit neurotransmitter release [31,32].

To clarify how 5-HT3Rs modulate the signaling between primary afferent fibers and dorsal horn neurons, in the present study, we describe, in newborn and adult rats, different aspects of serotonergic signaling. We show variable functional 5-HT3Rs expression during development and describe the modulation of the glutamatergic spontaneous synaptic transmission between DRG and spinal neurons in co-culture due to direct Ca^2+^ entry through 5-HT3Rs. Although these receptors are known to be Ca^2+^-permeable [33,34], no experiments have yet been performed to quantify the Ca^2+^ permeability of 5-HT3Rs expressed by DRG neurons. Specifically, the fractional Ca^2+^ current (Pf, the percentage of total current carried by Ca^2+^ ions [35]) has been measured for the rat homomeric 5-HT3_A_ receptor stably expressed in HEK293 cells [36], as well for human recombinant 5-HT3_A_ and 5-HT3_AB_ receptors [37], but not in native cells. To support the view that Ca^2+^ influx through 5-HT3Rs is sufficient to account for the observed enhancement of transmitter release, we measured the Pf value of the 5-HT3Rs expressed by native DRG neurons, along with that of human 5-HT3_A_ and 5-HT3_AB_ receptors, for comparison and control.

## 2. Materials and Methods

### 2.1. Primary Neuronal Cultures

Cells were obtained from Wistar rats. Animals were used following the requirements of European Directive 86/609/EEC and the Ministry of Italian Health: Comm.21-6-12 (D.lgs.116/92) and aut. n° 987-8/2015-PR (D.lgs. 26/2014). All rats were anesthetized before killing. Spinal neurons were taken from young postnatal rats (p1–3). The vertebral column was dorsally opened, and the cord was removed to a dish containing phosphate-buffered saline (PBS). Columns from four or five animals were combined and incubated for 30–90 min in oxygenated Earl’s buffer containing papain. The cells were dissociated by trituration after rinsing several times with Earl’s buffer containing BSA, 1 mg/mL. Adult (p20–30) or neonatal (p1–3) acutely dissociated DRG neurons were prepared as previously reported [38]. Briefly, thoracic and lumbar ganglia were excised and placed into PBS. DRGs were then minced and incubated with an enzymatic mix containing collagenase (1.0 mg/mL) and DNase (0.1 mg/mL) at 37 °C for 25 min. Dissociated DRG neurons were plated either alone or together with spinal neurons. Dissociated cells were plated onto 35 mm culture dishes coated with a mixture of poly-D-ornithine (0.2 mg/mL) and laminin (6 mg/mL). The cultures were maintained at 37 °C in a humidified, 5% CO_2_ incubator, in Eagle’s minimal essential medium supplemented with 20 mM glucose, 0.5 mM glutamine, 100 U/mL penicillin, 0.1 mg/mL streptomycin, and 4% horse serum. The adult DRG neurons were used 24 h after plating.

In co-cultures of newborn DRG and spinal neurons, obtained from postnatal days p1–3 animals, we were able to identify typical DRG neurons and spinal cord neurons by their morphological and electrophysiological characteristics. Specifically, multipolar and spindle-like cells with well-developed dendrites were identified as spinal cord neurons. In contrast, DRG neurons were much larger than spinal cord neurons and had a typical oval pseudounipolar shape. In addition, some DRG neurons displayed round somata and two processes running from the soma poles or, alternatively, were rounded cells having a thin axon and several short-ramified dendrites. The electrophysiological criterion for identifying spinal neurons was the presence of recordable synaptic inputs in spinal neurons, whereas DRG neurons completely lacked this kind of activity [39]. The co-cultures were treated with 2 μM cytosine β-D-arabinofuranoside and were used for experiments between 7 and 15 days in vitro.

The majority of recorded DRG neurons displayed small size (21 ± 2 pF and 37 ± 3 pF for newborn and adult DRG respectively) and were sensitive to capsaicin (85% of newborn and 90% of adult neurons).

### 2.2. Expression of Human 5-HT3_A_ and 5-HT3_B_ in GH4C1 Cells

The human 5-HT3_A_ and 5-HT3_B_ cDNAs were kindly provided by Dr. E. F. Kirkness (Institute for Genomic Research, Rockville, MD, USA) and were used to transiently transfect rat anterior pituitary GH4C1 cells, plated at a density of 5–10 × 10^4^ per 35-mm Petri dish, and grown in Ham’s F10 nutrient mixture, with 10% fetal bovine serum and 1% penicillin and streptomycin. Transient transfection was achieved by adding to each dish 1 μg of human 5-HT3 subunit cDNA, along with 4 μL of lipofectamine. All culture media were purchased from Invitrogen (Monza, Italy).

### 2.3. Electrophysiology

Whole-cell currents were recorded at room temperature from cells voltage-clamped, unless otherwise indicated, at −70 mV using borosilicate glass patch pipettes having a tip resistance of 3–5 MΩ. Membrane currents were filtered at 2 kHz upon acquisition with an Axopatch 200B amplifier (Axon Instruments, Foster City, CA, USA) and analyzed using pCLAMP 10 software (Axon). During recordings, the cells were continuously superfused using a gravity-driven perfusion system consisting of independent tubes for normal and drug-containing external solutions. The terminals of the tubes were positioned 50–100 µm away from the patched cell and the other ends were connected to a fast exchanger system (RSC-100; Bio-Logic, Seyssinet-Pariset, France).

### 2.4. Ca^2+^ Measurements

The methods for Ca^2+^ and Pf determinations are fully reported in [38]. The cells were incubated at 37 °C with the membrane-permeant fluorescent dye fura-2 AM (4 µM) for 45 min in DMEM. Fluorescence determinations were created using a conventional system driven by Axon Imaging Workbench software (Axon Instruments). All optical parameters and digital camera settings were maintained throughout this study to avoid non-homogeneous data. The recordings of the fluorescence signals and whole-cell membrane currents were synchronized, and images were acquired and stored on a PC and analyzed offline. The changes in intracellular free calcium concentration ([Ca^2+^]_i_) were expressed as ΔR (i.e., the difference between the peak and basal values of the ratio R between digital images acquired with two excitation wavelengths, 340 and 380 nm, monitoring emission at 510 nm), and ΔF/F (i.e., the ratio of time-resolved fluorescence variation over the basal fluorescence, using only one excitation wavelength, 380 nm). Specifically, the ΔF/F ratio was used when determining the Pf value, increasing the temporal resolution. Electrodes were filled with intracellular solution (see below) containing cell-impermeant fura-2 (250 µM; Molecular Probes). Determinations were carried out after the basal fluorescence had reached a stable value. The cells displaying high basal F_340_/F_380_ ratio values (>2 in our conditions) and/or low basal F_380_ values (<100 a.u.) were discarded. To evaluate Pf, the F/Q ratio between the fluorescence increase (F) and the total charge that had entered the cell at each fluorescence acquisition time (Q) was defined as

F/Q = (ΔF/F)/∫ I dt

For each cell, we used the F/Q points that, immediately after the onset of the m-chlorophenyl-biguanide (mCPBG)-induced response, exhibited a linear relationship, indicating that the Ca^2+^-buffering capability of fura-2 was not saturated. The F/Q ratio value was then measured as the slope of the linear regression best fitting the F–Q plot. Finally, Pf was determined by normalizing the ratio obtained in standard medium (F/QS) to the calibration ratio, measured when Ca^2+^ ions were the only permeant ionic species (F/QCa):

Pf = (F/QS)/(F/QCa)

### 2.5. Solutions and Chemicals

Cells were superfused with a standard external medium containing (mM): NaCl, 140; KCl, 2.8; CaCl_2_, 2; MgCl_2_, 2; Hepes–NaOH, 10; glucose, 10; pH 7.3. The Ca^2+^-free solution contained EGTA, 2 mM. The standard intracellular solution for whole-cell recording contained (mM): CsCl, 140; Hepes–CsOH, 10; Mg_2_ATP_3_, 2; and BAPTA, 5; pH 7.3. F/Q calibrations were performed in a medium (calibration medium) containing Ca^2+^ as the only permeant ion (mM): N-methyl-D-glucamine, 142; CaCl_2_, 2; and Hepes–Ca(OH)_2_, 10, pH 7.3; or N-methyl-D-glucamine, 130; CaCl_2_, 10; and Hepes–Ca(OH)_2_, 10, pH 7.3. For all the measurements of Pf, patch-clamp electrodes were filled with an internal solution containing (mM): N-methyl-D-glucamine, 140; Hepes–HCl, 10; fura-2, 0.25; thapsigargin, 0.001, pH 7.3. All chemicals were purchased from Sigma-Aldrich (Saint Louis, MO, USA), except for fura-2 and fura-2 AM, from Molecular Probes (Eugene, OR, USA), and for bicuculline and mCPBG, from Tocris (Bristol, UK).

### 2.6. Data Analysis and Statistics

Data are reported as means ± SEM, and statistical significance is tested using ANOVA (*p* < 0.05). The F/Q ratio values used in the Pf determinations were obtained as linear regressions of the data, using SigmaPlot software (version 14, Systat Software Inc., Chicago, IL, USA). Spontaneous miniature excitatory postsynaptic currents (mEPSCs) were analyzed with the Mini Analysis program (Synaptosoft). The mEPSCs were automatically identified by the software as trace deflections crossing area and amplitude thresholds, and then inspected by eye to avoid misclassification. The statistical significance of cumulative probability curves was assessed using the Kolmogorov–Smirnov test.

## 3. Results

### 3.1. 5-HT3 Receptor-Mediated Responses in DRG Neurons

**Figure 1:**
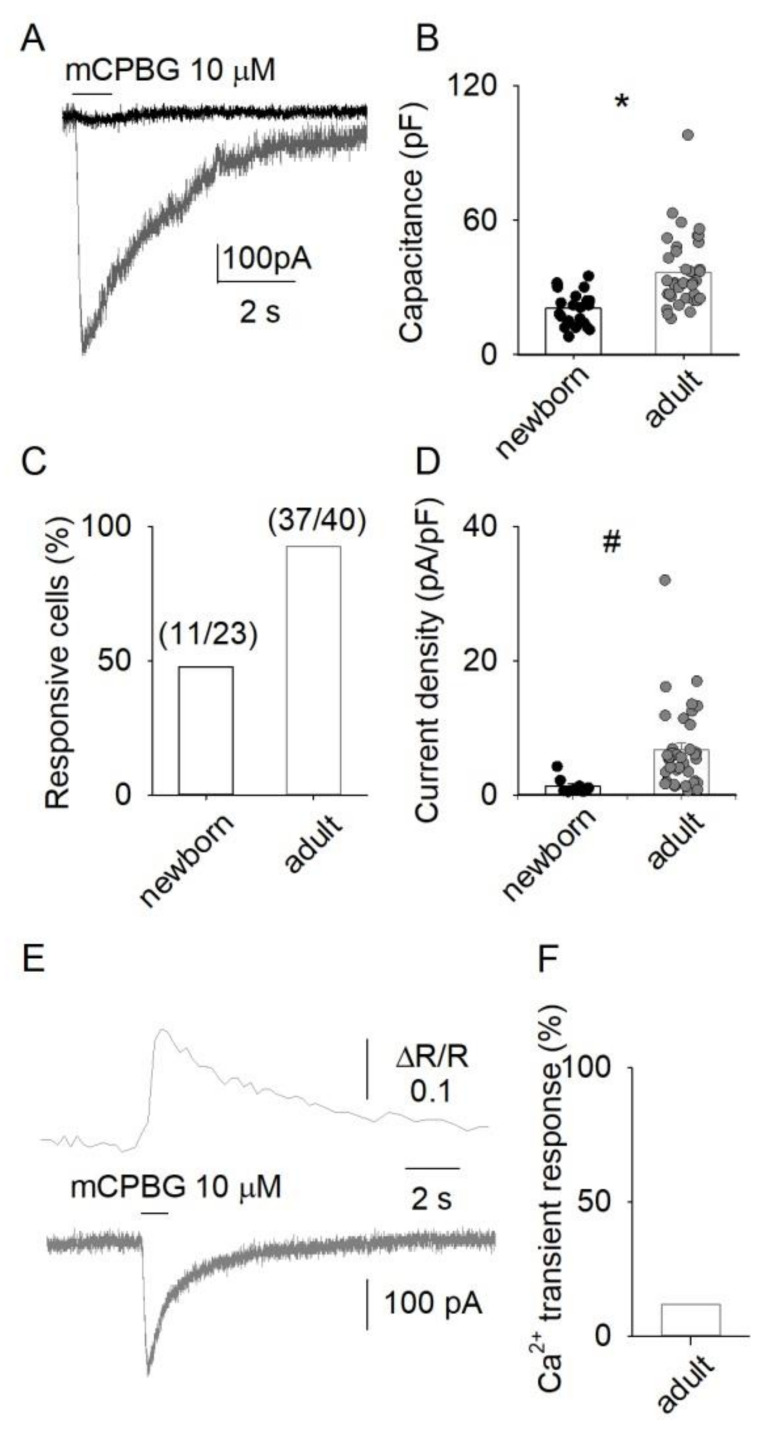
The activation of 5-HT3Rs evokes inward whole-cell currents in DRG neurons. (**A**) Superimposed traces showing the currents evoked by 1 s application of mCPBG 10 μM in DRG neurons isolated from a newborn rat (black line) or adult rat (dark-grey line). (**B**) Bar graph representing capacitance mean values obtained from newborn or adult DRG cells (* *p* < 0.001, F = 18.8). (**C**) percentage of cells responding to mCPBG with an inward current, in newborn or adult. (**D**) Bar graph illustrating the mean current density values in responsive newborn or adult DRG cells (#, *p* = 0.007, F = 7.5). (**E**), Typical recordings of the [Ca^2+^]_i_ increase (**top**) and of the inward current (**bottom**; holding potential, −70 mV) elicited by the application of 10 μM mCPBG to two different neurons. (**F**) Percentage of adult DRG neurons responding to 10 μM mCPBG with an increase in [Ca^2+^]_i_; please note the lower responsiveness in Ca^2+^ in comparison with transmembrane current (panel **C**, **right**).

The application of mCPBG (10 μM), a selective agonist of 5-HT3Rs [39], evoked inward whole-cell currents in 92.5% (37 out of 40) of the adult DRG neurons examined, with a mean amplitude of −223 ± 32 pA (Figure 1A–D; n = 37) and a mean current density of 7 ± 1 pA/pF. By contrast, in 52.2% of the newborn DRG cells (11 out of 23), we found small inward currents of −25 ± 5 pA (current density of 1.3 ± 0.4 pA/pF; Figure 1A–D; n = 11). These findings indicate that in DRG neurons functional 5-HT3Rs expression is low in the early post-natal life and increases in adult life. Moreover, mCPBG evoked detectable Ca^2+^ transients in 12% of the adult DRG neurons (16 out of 136 cells; Figure 1E,F), showing a mean amplitude (ΔR) value of 0.29 ± 0.13. These findings indicate that DRG neurons widely express functional 5-HT3Rs, whose activation produces detectable [Ca^2+^]_i_ elevations only in a subset of cells, suggesting that these receptors have a relatively low Ca^2+^ permeability.

### 3.2. Ca^2+^ Permeability of Somatic 5-HT3Rs in Adult DRG Neurons

**Figure 2:**
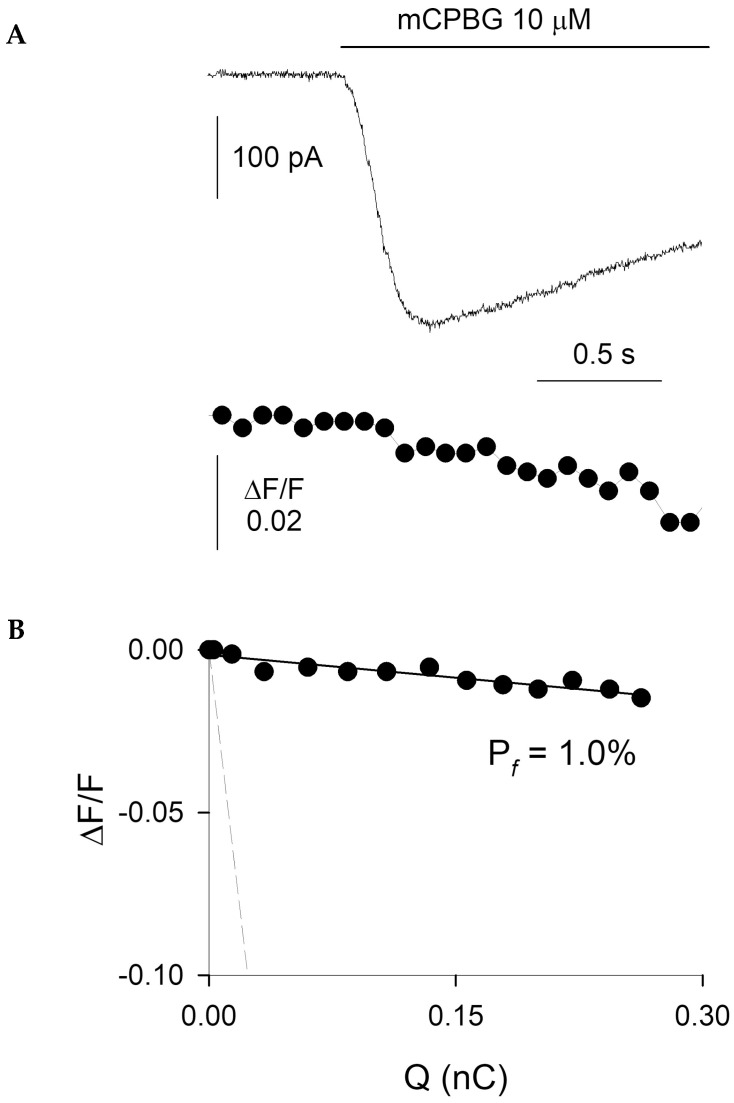
Ca^2+^ permeability of 5-HT3Rs expressed in native DRG neurons. (**A**) Simultaneous recordings of the whole-cell current (top, holding potential, −70 mV) and fluorescence transient (bottom) elicited by mCPBG as indicated in a DRG neuron equilibrated in standard medium. Current and fluorescence traces are aligned, sharing the same time scale. (**B**) Linear relationships between ΔF/F (i.e., Ca^2+^-dependent fluorescence variation) and Q (total electric charge) obtained from the same cell. For this example, the value of the F/QS ratio is 0.045 nC-1, while the value of the F/QCa ratio (slope of the dotted line) is 4.3 nC-1, yielding a Pf value of 1.0%.

**Figure 3:**
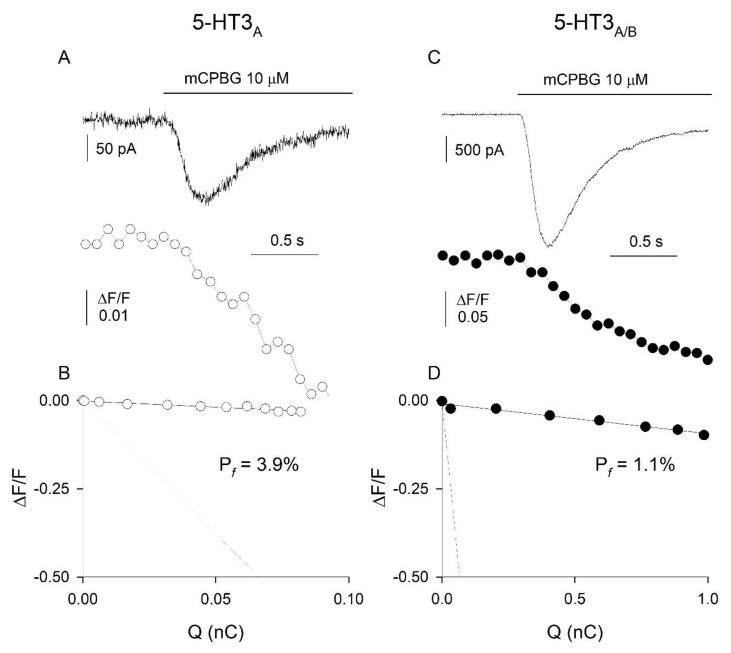
Ca^2+^ permeability of homomeric 5-HT3_A_ and heteromeric 5-HT3_A_/3_B_ receptors. (**A**) Simultaneously recorded 5-HT3-mediated inward current (**top**) and Ca^2+^ transient (**bottom**), and (**B**) Linear relationships as in Figure 2, from a GH4C1 cell transiently transfected with 5-HT3_A_ subunit cDNA. Time scale bar is shared for current and Ca^2+^ traces. For this example, the value of the F/QS ratio is 0.296 nC-1, while the value of the F/QCa ratio (slope of the dotted line) is 7.5 nC-1, yielding a Pf value of 3.9%. (**C**,**D**) Recordings, as in (**A**,**B**), from a GH4C1 cell transiently transfected with the human 5-HT3_A_ and 5-HT3_B_ subunit cDNAs. For this example, the value of the F/QS ratio is 0.084 nC-1, yielding a Pf value of 1.1%. Concentration and applications of mCPBG as indicated.

Given that Ca^2+^ entry at presynaptic terminals represents a major determinant of synaptic neurotransmitter release, it is relevant to know the Ca^2+^ permeability of presynaptic DRG 5-HT3Rs. However, the direct measurement of the Pf of presynaptic 5-HT3Rs is not feasible, due to improper electrical clamp and internal dialysis. Thus, we analyzed the Pf of somatic 5-HT3Rs expressed in adult rat cultured DRG neurons (which are deprived of processes, thus allowing proper space clamp and internal dialysis) by simultaneously recording both mCPBG-induced inward currents and Ca^2+^ transients (Figure 2A). The mean F/Q ratio value obtained in a standard medium was 0.044 ± 0.004 nC-1 (n = 7), while the mean F/Q ratio value corresponding to a Pf of 100% (current carried only by Ca^2+^ ions) was 4.3 ± 0.3 nC-1 (n = 16; not shown). The ratio between the F/Q values obtained in the standard and the calibration media yielded a Pf value of 1.0 ± 0.2% (n = 7; Figure 2B). It has been previously reported that the Pf of recombinant rat homomeric 5-HT3_A_Rs is, on average, 4.7% [28], a value four-fold larger than the Pf value here reported for native rat DRG neurons. To compare the Ca^2+^ permeability of 5-HT3Rs expressed in DRG neurons with that of 5-HT3Rs formed with a known receptor subunit composition, we measured the Pf values of both homomeric 5-HT3_A_Rs and heteromeric 5-HT3_A/B_Rs, expressed by transient transfection in the pituitary GH4C1 cell line, an expression cell system suitable for Pf determinations [40]. The Pf values were 3.5 ± 1.0% (n = 5) and 1.1 ± 0.2% (n = 8) for 5-HT3_A_Rs and 5-HT3_A/B_Rs, respectively (Figure 3A–D). Thus, the Ca^2+^ permeability of heteromeric 5-HT3Rs matches the Ca^2+^ permeability of native somatic DRG 5-HT3Rs, supporting the view that the latter receptors are heteromeric and composed of 5-HT3_A_ and 5-HT3_B_ subunits [18,28].

### 3.3. Modulatory Role of 5-HT3Rs in Neurotransmission

**Figure 4:**
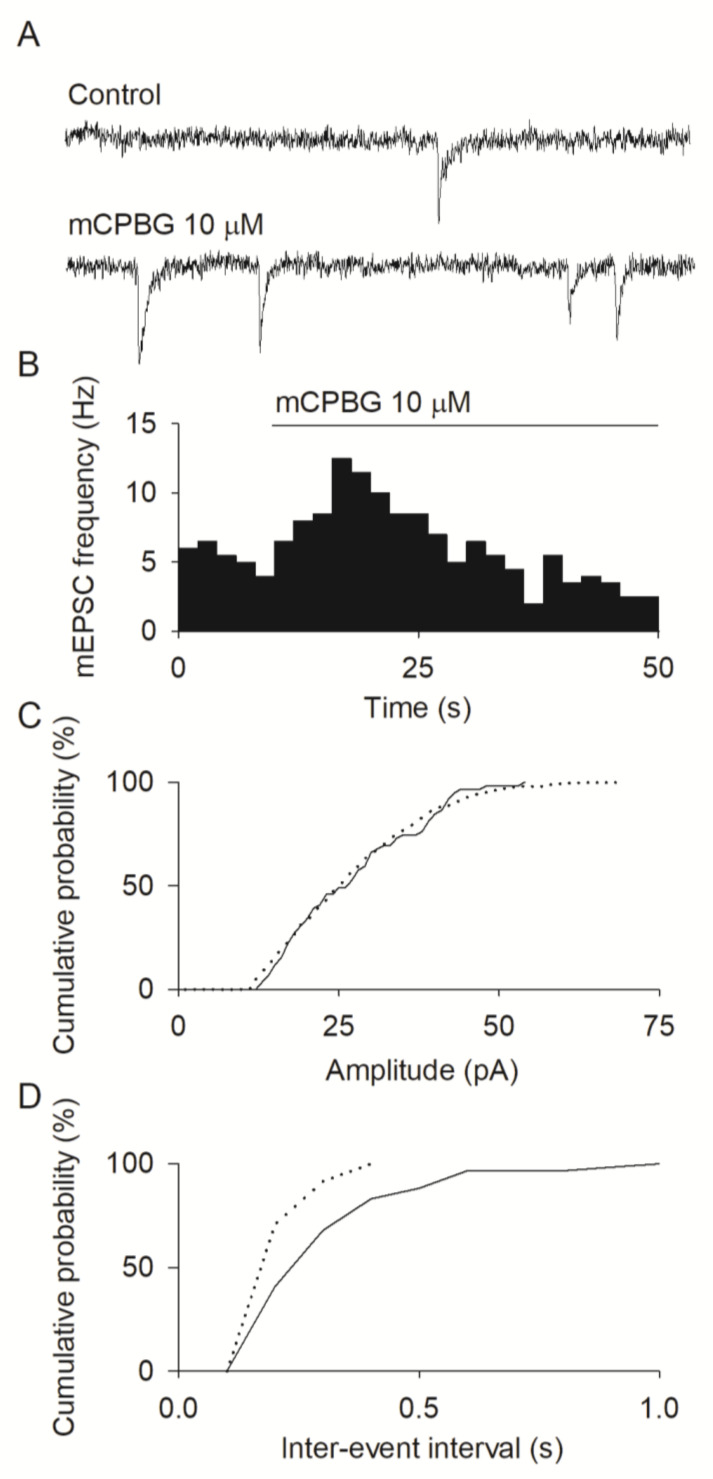
5-HT3Rs modulate the spontaneous synaptic transmission between neonatal DRG and spinal neurons in co-culture. (**A**) Whole-cell recording from a spinal neuron in co-culture with DRG neurons showing mEPSCs in control conditions (**top**) and during application of mCPBG at the indicated concentration (bottom). The mCPBG-induced frequency increase was observed in 12 out of 29 cells. Lidocaine (10 mM), bicuculline (10 μM), and strychnine (100 nM) were present in a Mg^2+^-free medium. Holding potential, −70 mV. (**B**) Time course of mEPSC frequency (2 s/bin) for the same recording as (**A**). (**C**,**D**) Cumulative distribution of either mEPSC amplitudes or time intervals separating mEPSC events before (solid line) and during (dotted line) the application of mCPBG. Control and mCPBG cumulative distribution of inter-event intervals are significantly different (Kolmogorov-Smirnov test, *p* = 0.003).

To investigate the physiological role of 5-HT3Rs expressed by DRG neurons, and to test whether these receptors may affect the signaling between sensory and spinal neurons, we analyzed the effects of their activation on the spontaneous neurotransmitter release from DRG presynaptic terminals. For this purpose, we focused on co-cultures of neonatal DRG and spinal neurons, recording the spontaneous mEPSCs in spinal neurons, exposed to lidocaine (10 mM) to block voltage-activated sodium channels [41], to bicuculline (10 μM) along with strychnine (100 nM) to eliminate inhibitory neurotransmission, and to Mg^2+^-free medium to allow NMDA receptor activation. In co-cultures, the application of mCPBG (10 μM) evoked inward currents in six out of a total of nineteen DRG neurons examined (not shown), while 37 spinal neurons examined did not exhibit inward currents evoked by mCPBG (not shown). In agreement with these findings, there was again a lack of current response to mCPBG in spinal monocultures (two experiments, 10 neurons examined; not shown). In the same neurons from spinal monocultures, the application of mCPBG also did not affect mEPSC frequency or amplitude. In contrast, the application of mCPBG (10 μM) transiently increased the mEPSC frequency by 400 ± 100% in 12 out of the total 29 spinal neurons examined in co-culture (Figure 4A,B), leaving unaffected the mEPSC amplitudes (Figure 4C,D). Considered together, these findings suggest that, under our experimental conditions, cultured spinal neurons did not express functional 5-HT3Rs, and that the mCPBG-induced enhancement of frequency observed in spinal neurons co-cultured with DRG neurons was genuinely due to the activation of 5-HT3Rs expressed by DRG neurons.

### 3.4. Modulatory Role of Ca^2+^ Entry through 5-HT3Rs

**Figure 5:**
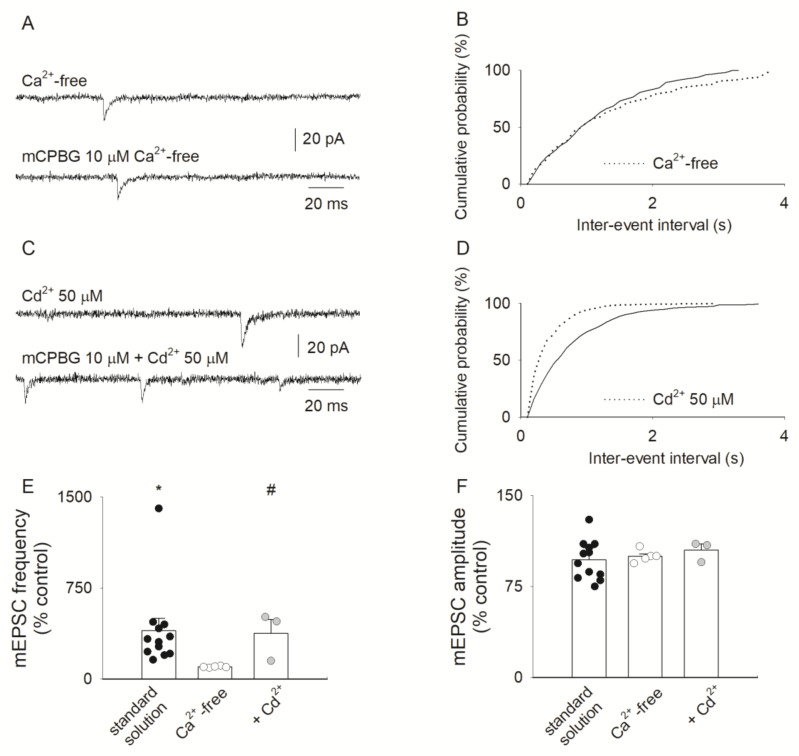
The 5-HT3Rs-mediated modulation of mEPSCs depends on external Ca^2+^ and is independent of voltage-activated Ca^2+^ channels. (**A**) Whole-cell recordings of mEPSCs from a spinal neuron in co-culture with DRG neurons in a Ca^2+^-free medium before (**top**) and during (**bottom**) application of 10 μM mCPBG; typical of five experiments. (**B**) Cumulative distribution of the time interval separating mEPSC events in Ca^2+^-free medium before (solid line) and during (dotted line) the application of mCPBG. (**C**) mEPSC recordings from a different spinal neuron in a medium containing 50 μM Cd^2+^ before (**top**) and during (**bottom**) application of 10 μM mCPBG. (**D**) Plot as B, in the presence of 50 μM Cd^2+^, before (solid line) and during (dotted line, *p* < 0.05) the application of mCPBG. (**E**) Histogram representing the mean mEPSC frequency from spinal neurons exposed to mCPBG (10 μM) in normal external solution (black circles, n = 12; * *p* = 0.005, F = 9.6), in Ca^2+^-free medium (white circles, n = 5) or in 50 μM Cd^2+^-containing medium (gray circles, n = 3; # *p* = 0.016, F = 11.0). Note Ca^2+^ withdrawal inhibiting mCPBG-induced frequency enhancement. (**F**) Histogram showing unchanged mEPSC amplitudes in the same experiments as (**E**).

Experiments were carried out to investigate whether the Ca^2+^ influx through 5-HT3Rs could influence the mCPBG-induced enhancement of mEPSCs frequency in spinal neurons co-cultured with DRG neurons. Ca^2+^ withdrawal from the extracellular medium suppressed the mCPBG-induced increase in mEPSC frequency (e.g., Figure 5A,B; n = 5), indicating that Ca^2+^ entry triggered by the activation of 5-HT3Rs was a key step in promoting the neurotransmitter release.

To see whether Ca^2+^ entry through the voltage-activated Ca^2+^ channels (VACCs) could contribute to the modulation of neurotransmitter release mediated by 5-HT3Rs, we added to the external solution the VACC blocker Cd^2+^ (50 μM) [42]. Under these conditions, mCPBG still significantly increased the mEPSC frequency in three out of nine cells examined (e.g., Figure 5C,D), in agreement with findings obtained in the absence of Cd^2+^ (Figure 5E). Furthermore, mCPBG in the presence of Cd^2+^ did not alter significantly the mEPSC amplitude (Figure 5F). Considered together, all these findings indicate that Ca^2+^ entry through 5-HT3 receptor channels, rather than through the subsequent activation of VACCs, plays a presynaptic modulatory role in spontaneous synaptic neurotransmission.

## 4. Discussion

We described different aspects of 5-HT3Rs function in rat primary afferent cells, showing differential expression in newborn and adult DRG neurons. Also, we quantified the Ca^2+^ influx through 5-HT3Rs expressed by adult DRG neurons, showing that somatic receptors display the lowest permeability to Ca^2+^ in the superfamily of ionotropic pentameric ligand-activated receptors reported to date (reviewed in [43,44]). This pattern is probably determined by the heteromeric composition of the native 5HT3Rs, whose Ca^2+^ permeability is similar to that of recombinant heteromeric receptors, composed of 5-HT3_A/B_ subunits, while it is much lower than that of recombinant homomeric receptors composed of 5-HT3_A_ subunits. Finally, we demonstrated that the Ca^2+^ influx through 5-HT3_A/B_ receptors induces an enhancement of the excitatory mEPSCs frequency at synapses between co-cultured DRG and spinal neurons, used as a model of first somatosensory synapses.

### 4.1. Expression of 5-HT3Rs in DRG Neurons

Many studies have analyzed the effects of 5-HT on DRG neurons but the majority of them focused on the metabotropic 5-HT receptors and only a few on the pathophysiological role of the 5-HT3Rs. Specifically, the receptor is found in both myelinated and unmyelinated nociceptors, being pronociceptively involved in persistent, but not acute, pain signaling [29]. The vast majority of DRG neurons analyzed in the present study are represented by nociceptors, as suggested by their small size and high capsaicin sensitivity. The 5-HT3R promotes depolarization and Ca^2+^ influx in rat DRG neurons [8,45] and contributes to the excitation of rat colonic afferent fibers [27]. Interestingly, inflammatory pain seems to be able to positively modulate the expression of the 5-HT3Rs in DRG neurons [46]. The mRNA coding for the 5-HT3_A_ subunit has been detected in most DRG neurons (70%), 20–40% of which are reported to also express the 5-HT3_B_ subunit [6,28]. We report here a marked difference in the percentages of adult DRG cells exhibiting mCPBG-induced currents (~82%) or Ca^2+^ transients (~12%). This apparent discrepancy is probably due to the low 5-HT3-mediated current density observed in DRG neurons, unable to produce a detectable elevation in [Ca^2+^]_i_ in most cells. Furthermore, the functional expression of 5-HT3Rs appears lower in neonatal than in adult DRG neurons. This finding suggests a developmental control of 5-HT3 expression.

### 4.2. Presynaptic 5-HT3Rs in DRG Neurons

The 5-HT3Rs of DRG neurons have been localized both in peripheral nerve endings [47,48,49] and in the central terminals targeting the superficial layers of the spinal dorsal horn [25,30,50]. However, while the functional effects of the activation of 5-HT3Rs on peripheral fibers have been described [27,29], our data give the first evidence that 5-HT3Rs can facilitate the release of glutamate from presynaptic terminals at sensory–spinal synapses. Our findings are in agreement with previous studies showing the 5-HT3-mediated facilitation of the release of sensory neuropeptides from primary afferents in rat spinal cords [51,52]. Similar facilitatory presynaptic mechanisms are documented for 5-HT3Rs in other CNS regions [19,20,21,22,23,24,25,26].

Interestingly, although 5-HT3Rs have been localized in spinal interneurons [53,54,55], in our co-cultures we could not find any inward current induced by mCPBG in spinal neurons. This finding supports the hypothesis that mCPBG enhances the frequency of mEPSCs recorded from spinal neurons by acting on 5-HT3Rs present at presynaptic DRG terminals. In this study we have also reported that the mCPBG-induced increase in mEPSC frequency depends on the Ca^2+^ influx being completely blocked following the withdrawal of Ca^2+^ from the external medium and is independent of VACCs as it is not significantly affected by Cd^2+^, the latter indicating that the activation of VACCs is not necessary for modulating the synaptic activity, as for the 5-HT3-induced facilitation of GABA release in the hippocampus [56].

### 4.3. Ca^2+^ Permeability of 5-HT3Rs

The ability of Ca^2+^ ions to flow through 5-HT3 receptor channels has long been known [33,34], along with the evidence that their Ca^2+^ permeability is relatively low [57,58]. This view is confirmed by our data, providing for the first time the Pf value (Pf~1%) for native 5-HT3Rs. This value is very similar to that here measured for the human heteromeric 5-HT3_A/B_Rs (Pf = 1.1%) but lower than homomeric 5-HT3_A_Rs (Pf = 3.5%), supporting the view that DRG neurons functionally express both 5-HT3 subunits [28]. Our findings with human recombinant receptors are in agreement with a previous study reporting Pf values of 2.0% and 4.1% for homomeric and heteromeric human 5-HT3Rs expressed in HEK293 cells [37] and with earlier findings showing that the permeability ratio PCa/PCs of human homomeric 5-HT3_A_Rs (~1) is higher than that of heteromeric 5-HT3_A/B_Rs (~0.6), indicating that the insertion of the 5-HT3_B_ subunit produces a drop in the Ca^2+^ permeability of 5-HT3Rs [15,59]. The Pf value of the rat homomeric 5-HT3_A_ receptor stably expressed in HEK293 cells, 4.7% [36], is not far from the value of the human 5-HT3_A_ receptor, indicating that the Ca^2+^ permeability properties of 5-HT3Rs are maintained in different species. We measured the Pf value of 5-HT3Rs expressed in the soma of adult DRG neurons lacking neurites. This experimental condition, necessary for attaining both proper internal dialysis and correct voltage clamp, must be taken into account when evaluating the physiological role of the 5-HT3Rs in these cells. For instance, it is known that in rat striatum, perykarial 5-HT3Rs are less Ca^2+^-permeable than those at presynaptic sites [20]. However, to date, there is no evidence suggesting a possible differential subunit composition of 5-HT3Rs located in different regions of DRG neurons.

## 5. Conclusions

This paper points to an important neuromodulatory role of Ca^2+^ entry mediated by presynaptic 5-HT3Rs, contributing to the elucidation of the complex role of 5-HT in peripheral and spinal mechanisms of sensory processing, and adds another value to the list of Pf values for ligand-gated receptors [40,43,44]. We speculate that the 5-HT released at the spinal cord from bulbospinal projections (see for review [60]) probably also binds to 5-HT3Rs expressed in the terminals of primary afferents, producing a positive modulation of the neurotransmission between DRG and spinal neurons. This mechanism may also underlie the 5-HT3-mediated facilitation of the mechanical allodynia observed after spinal cord injury [61].

## Data Availability

Relevant data are contained within the article.
